# Indole Sensing Regulator (IsrR) Promotes Virulence Gene Expression in Enteric Pathogens

**DOI:** 10.1128/mbio.01939-22

**Published:** 2022-08-02

**Authors:** Aman Kumar, Regan M. Russell, Mehmet Ali Hoskan, Vanessa Sperandio

**Affiliations:** a Department of Microbiology, University of Texas Southwestern Medical Center, Dallas, Texas, USA; b Department of Biochemistry, University of Texas Southwestern Medical Center, Dallas, Texas, USA; University of Georgia

**Keywords:** enterohemorrhagic *E. coli* (EHEC), *Citrobacter rodentium*, indole, locus of enterocyte effacement (LEE)

## Abstract

Enteric pathogens such as enterohemorrhagic E. coli (EHEC) and its surrogate murine model Citrobacter rodentium sense indole levels within the gut to navigate its biogeography and modulate virulence gene expression. Indole is a microbiota-derived signal that is more abundant in the intestinal lumen, with its concentration decreasing at the epithelial lining where it is absorbed. E. coli, but not C. rodentium, produces endogenous indole because it harbors the *tnaA* gene. Microbiota-derived exogenous indole is sensed by the CpxAR two-component system, where CpxA is a membrane-bound histidine-sensor-kinase (HK) and CpxR is a response regulator (RR). Indole inhibits CpxAR function leading to decreased expression of the locus of enterocyte effacement (LEE) pathogenicity island, which is essential for these pathogens to form lesions on enterocytes. In our transcriptome studies comparing wild-type (WT) EHEC and Δ*tnaA* ± indole, one of the most upregulated genes by indole is *ygeV*, which is a predicted orphan RR. Because of the role YgeV plays in the indole signaling cascade, we renamed this gene indole sensing regulator (*isrR*). In the absence of endogenous indole, IsrR activates LEE gene expression. IsrR only responds to endogenous indole, with exogenous indole still blocking virulence gene expression independently from IsrR. Notably, a C. rodentium *isrR* mutant is attenuated for murine infection, depicting delayed death, lower intestinal colonization, and LEE gene expression. IsrR aids in discriminating between microbiota-derived (exogenous) and endogenous self-produced indole in fine-tuning virulence gene expression by enteric pathogens in the intestine.

## INTRODUCTION

The mammalian gut has a rich chemistry landscape derived from both the host and the microbiota, influencing the biogeography of the gastrointestinal (GI) tract. Enteric bacterial pathogens sense and respond to these info-chemicals in their environment in a manner that culminates in the most spatiotemporal efficient expression of their virulence genes (1). The colon contains tryptophan derivatives, including the microbiota-derived indole that is more abundant in the luminal compartment, where the microbiota resides, and is depleted at the epithelial lining because it is absorbed by the epithelium (2, 3). Indole is synthesized by tryptophanase that is encoded by the *tnaA* gene. Both E. coli and other members of the gut microbiota produce indole (2).

Indole, at physiological concentrations found within the GI tract, decreases the expression of the virulence genes in the enteric pathogens EHEC and C. rodentium (2), a murine pathogen extensively employed as a surrogate animal model for EHEC (4, 5). EHEC colonizes the human colon, leading to outbreaks of bloody diarrhea and hemolytic uremic syndrome (HUS) worldwide (6). EHEC virulence determinants include the production of the potent Shiga toxin (Stx) that causes HUS, and genes necessary for the attaching and effacing (AE) lesion formation on enterocytes. AE lesion formation requires genes contained within the locus of enterocyte effacement (LEE) pathogenicity island (PI) (7). The LEE region contains five major operons: *LEE1* to *LEE5* (8), which encode the Ler transcriptional activator of all LEE genes (8), a type III secretion system (T3SS) (9), an adhesin (10) and its receptor (11), and effector proteins (12).

The membrane-bound histidine kinase (HK) CpxA has been shown to be a receptor for the tryptophane derivatives serotonin (host neurotransmitter) and indole (2, 13). HKs can function as both kinases and phosphatases. CpxA autophosphorylates and phosphorylates the CpxR RR that directly binds to the regulatory region of *ler* to activate LEE gene expression. Upon sensing serotonin and/or indole, CpxA functions primarily as a phosphatase, dephosphorylating itself and CpxR, consequently blocking the activation of the LEE genes, leading to their decreased expression (2, 13). Using the murine microbiota-depleted C. rodentium infection model (5), it was also shown that self-produced or microbiota-derived indole reduces the expression of virulence genes, as well as C. rodentium virulence in mice (2). Indole in E. coli is synthesized by the tryptophanase enzyme, which converts L-tryptophan into indole (14) and is encoded by the *tnaA* gene. However, C. rodentium lacks the TnaA enzyme and cannot produce its own indole. An engineered C. rodentium with the EHEC *tna* operon inserted within its genome produces indole and is attenuated for murine infection (2).

Although both serotonin and indole decrease LEE expression, they differ in the regulation of *stx*. Serotonin does not affect *stx* expression, while indole decreases it (2, 13). This suggests that indole and serotonin have some overlapping targets, which may be mediated through the same receptor, CpxA in the case of the LEE, but also have differing targets that are indole specific, indicating that there is an indole specific receptor. Here we identified in our transcriptome studies that one of the most upregulated genes by indole is *ygeV*, which is a predicted orphan RR. We renamed YgeV indole sensing regulator (*isrR*) and showed that it activates the LEE in the absence of endogenous indole. IsrR is also important during C. rodentium murine infection with a Δ*isrR* mutant being attenuated. Here we identify a new member in the indole signaling cascade, adding to the complexity of this regulatory pathway.

## RESULTS

### Indole regulon in EHEC.

To have a global view of the indole regulon in EHEC, we previously performed RNAseq to compare the transcriptomes of wild-type (WT) EHEC and Δ*tnaA* EHEC with and without 500 μM indole (GEO accession no. GSE119440) (2). We identified several genes differentially regulated in response to indole in WT and Δ*tnaA*. A total of 1,110 genes were upregulated, and 1,374 genes were downregulated upon indole treatment in WT EHEC. Similarly, we found 1,156 upregulated and 1,311 downregulated genes upon indole treatment in the Δ*tnaA* background (Fig. S1A). Next, we identified the overlap in differentially expressed genes comparing WT and Δ*tnaA* with and without indole. We observed that a large number of genes were differentially regulated upon indole treatment in the two genotypes suggesting shared targets. We also observed enrichment of unique genes between the two genotypes in the presence or absence of indole (Fig. S1B).

10.1128/mbio.01939-22.1 FIG S1 Indole treatment differentially regulates several genes in EHEC. (A) Differential gene analysis using DESeq2 shows genes that are either significantly up or downregulated in response to indole. A cutoff of adjusted *P*-value <0.1 is considered significant. (B) Venn diagram comparing gene overlap between WT and Δ*tnaA* EHEC with and without indole. (C) Volcano plot indicating differentially regulated genes when comparing Δ*tnaA* + indole to Δ*tnaA* EHEC. *ygeV* (*isrR*) is identified as the gene significantly upregulated in response to indole. (A, and C) *P*-value is calculated using Wald’s test followed by multiple hypothesis correction using Benjamini-Hochberg to obtain adjusted *P*-values. Download FIG S1, TIF file, 0.9 MB. Copyright © 2022 Kumar et al. 2022 Kumar et al. https://creativecommons.org/licenses/by/4.0/ This content is distributed under the terms of the Creative Commons Attribution 4.0 International license.

**FIG 1:**
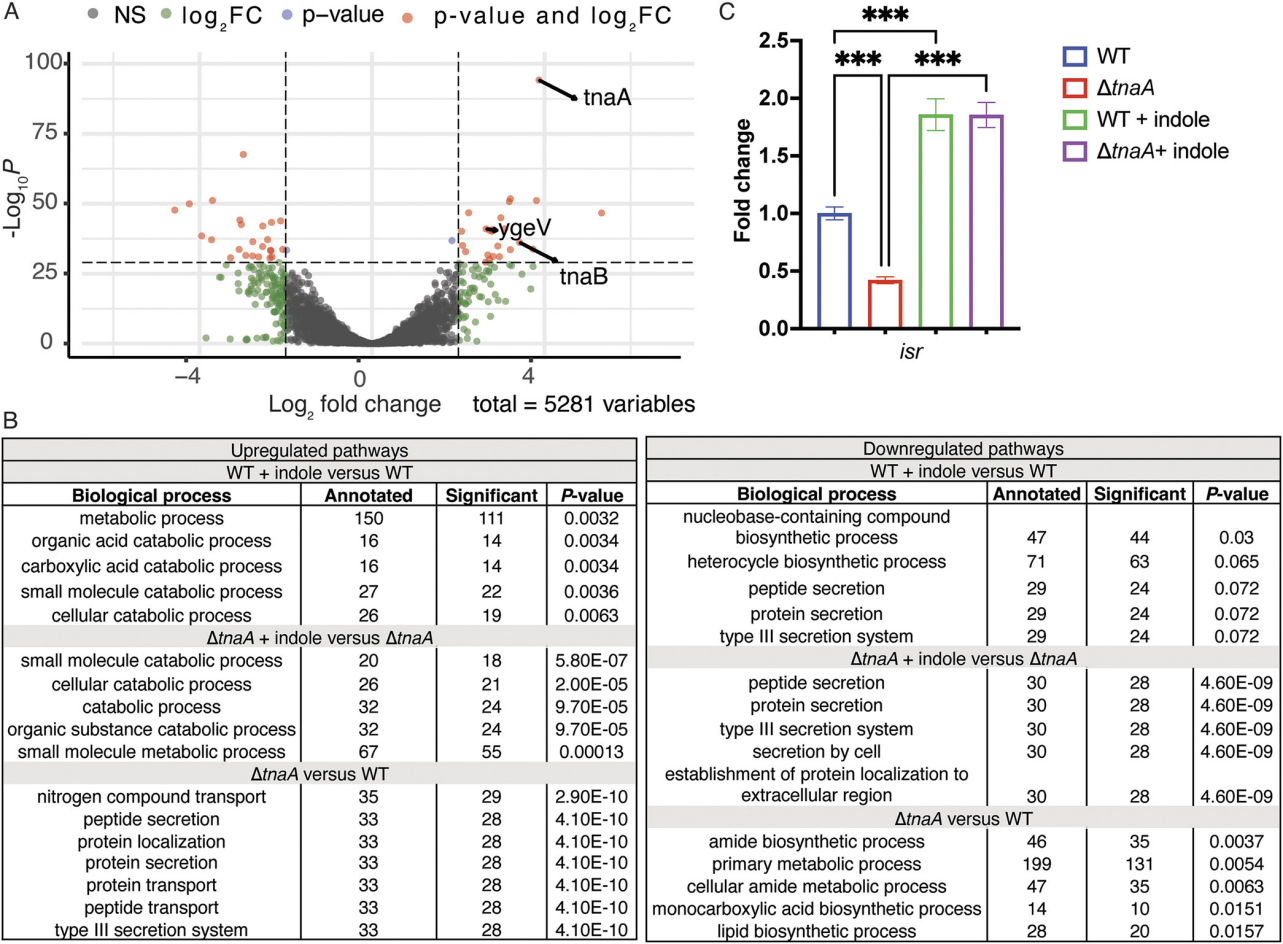
Indole increases expression of a putative transcriptional activator, *isrR*. (A) Volcano plot indicating differentially regulated genes when comparing WT + indole to WT EHEC. Genes that are significantly regulated with log_2_ fold change (log_2_FC) > 2 and with significant *P*-value are indicated in red. *ygeV* (*isrR*) is identified as the gene upregulated in response to indole. *P*-value is calculated using Wald’s test followed by multiple hypothesis correction using Benjamini-Hochberg to obtain adjusted *P*-values. (B) Gene ontology analysis, comparing WT and Δ*tnaA* with and without indole, showing the top five enriched pathways. *P*-value is determined using the Kolmogorov Smirnov test. (C) Quantitative real-time PCR analysis showing expression of *isrR* in response to indole. One-way ANOVA followed by Bonferroni’s multiple-comparison test is used to calculate the *P*-value. ***, *P < *0.001. Data are representative of at least two independent experiments with three biological replicates. Fold change was calculated relative to an internal control *rpoA*. Error bars represent standard deviations.

To identify pathways that are affected by indole treatment, we performed gene ontology analysis. We identified several altered metabolic pathways, notably, those involved in the metabolism of cyclic compounds were upregulated in the presence of indole. Indole treatment led to a decrease in the pathways related to protein secretion, virulence (type III secretion system), and biosynthesis of heterocyclic compounds (Fig. 1B). Predictably, virulence and secretion-related pathways were among the most differentially upregulated in Δ*tnaA* compared to WT, while metabolic and biosynthetic processes were downregulated in Δ*tnaA* (Fig. 1B). These observations are in line with our previous reports, where we showed that indole decreases the expression of genes implicated in virulence and secretion (2). One of the most upregulated genes upon indole treatment in both WT (Fig. 1A) and Δ*tnaA* (Fig. S1C) is an orphan RR encoded by the *ygeV* gene, which we renamed indole sensing regulator (*isrR*). IsrR is a putative σ^54^ dependent transcriptional activator consisting of a σ^54^ interaction domain as well as a helix-turn-helix DNA binding motif. Similar to commensal Escherichia coli, EHEC also harbors a *tna* operon consisting of two structural genes, *tnaA* encoding the tryptophanase enzyme required for indole production and *tnaB* encoding a low-affinity tryptophanase permease. Interestingly, we noticed a more robust effect of indole treatment on *isrR* expression in WT EHEC compared to Δ*tnaA* EHEC. The enriched expression of *isrR* may occur due to the additive effect of endogenous and exogenous indole present in WT EHEC. Indole is a quorum-sensing molecule (14), and EHEC increases the expression of tryptophanase in response to indole (Fig. S2). Therefore, as expected, the expression of *tnaA* and *tnaB* is significantly upregulated in response to indole within the RNAseq data set (Fig. 1A). Quantitative real-time PCR (qRT-PCR) confirmed that *isrR* is upregulated in response to indole in both WT and Δ*tnaA* EHEC (Fig. 1C).

10.1128/mbio.01939-22.2 FIG S2 Indole treatment increases expression of tryptophanase (*tnaA*) gene. qRT-PCR analysis comparing the expression of *tnaA* gene from WT EHEC in the presence of absence of indole. An unpaired student *t*-test is used to calculate statistics. **, *P < *0.01. Download FIG S2, PDF file, 0.03 MB. Copyright © 2022 Kumar et al. 2022 Kumar et al. https://creativecommons.org/licenses/by/4.0/ This content is distributed under the terms of the Creative Commons Attribution 4.0 International license.

### IsrR activates the expression of the LEE in the absence of the indole-producing enzyme, TnaA.

**FIG 2:**
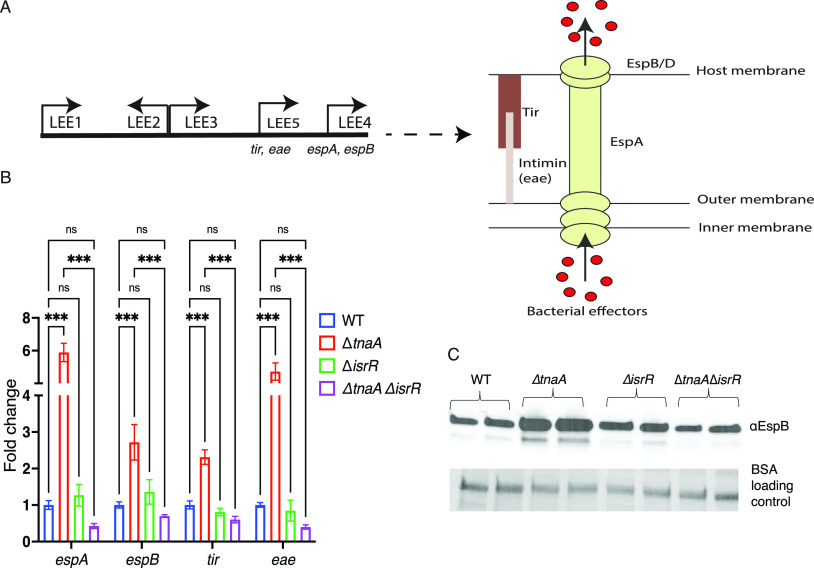
*isrR* activates LEE genes in the absence of indole-producing *tnaA*. (A) Schematic representation showing the LEE pathogenicity island and the representative virulence genes used for quantitative real-time PCR and Western blot analysis. The right scheme shows a cartoon representation of the type III secretion system, which is encoded by the LEE. (B) qRT-PCR analysis comparing expression of virulence-related genes in the WT, Δ*isrR*, Δ*tnaA*, and the double mutant Δ*tnaA*Δ*isrR* EHEC. *P*-value is calculated using Two-way ANOVA followed by Bonferroni’s multiple-comparison test. ***, *P < *0.001; ns, not significant. (C) Western blot comparison of the secreted protein EspB from WT, Δ*isrR*, Δ*tnaA*, and Δ*isrR*Δ*tnaA* EHEC grown anaerobically. BSA is used as a loading control. Data are representative of at least three independent experiments with three biological replicates. Fold change was calculated relative to an internal control *rpoA*. Error bars represent standard deviations.

EHEC senses and integrates microbial and host-derived signals to colonize the gut (15). EHEC attaches to enterocytes by expressing the LEE-encoded type III secretion system (Fig. 2A) (7, 9). Expression of the type III secretion system is an energy-expensive process, and therefore, EHEC fine-tunes its expression in response to several signaling molecules and regulators. To determine whether *isrR* is involved in EHEC virulence gene regulation, we constructed an Δ*isrR* EHEC. Expression of the LEE genes (*espA*, *espB*, *tir*, and *eae*) was similar between Δ*isrR* and WT EHEC (Fig. 2B). Because *isrR* is overexpressed in the presence of indole (Fig. 1), we constructed a Δ*tnaA*Δ*isrR* mutant, which is an *isrR* EHEC mutant that cannot produce indole. This allowed us to investigate *isrR*-dependent regulation of virulence in the absence of endogenously-produced indole. In contrast to Δ*isrR*, a Δ*tnaA*Δ*isrR* mutant is attenuated and expresses basal level of virulence genes compared to a Δ*tnaA* mutant (Fig. 2B). This suggested that *isrR* acts as an activator of virulence genes in the absence of the indole-producing tryptophanase enzyme. Congruent with the qRT-PCR expression data of virulence genes, there is also a decrease in the secretion of EspB, a LEE-encoded protein, in the double mutant compared to Δ*tnaA* (Fig. 2C).

**FIG 3:**
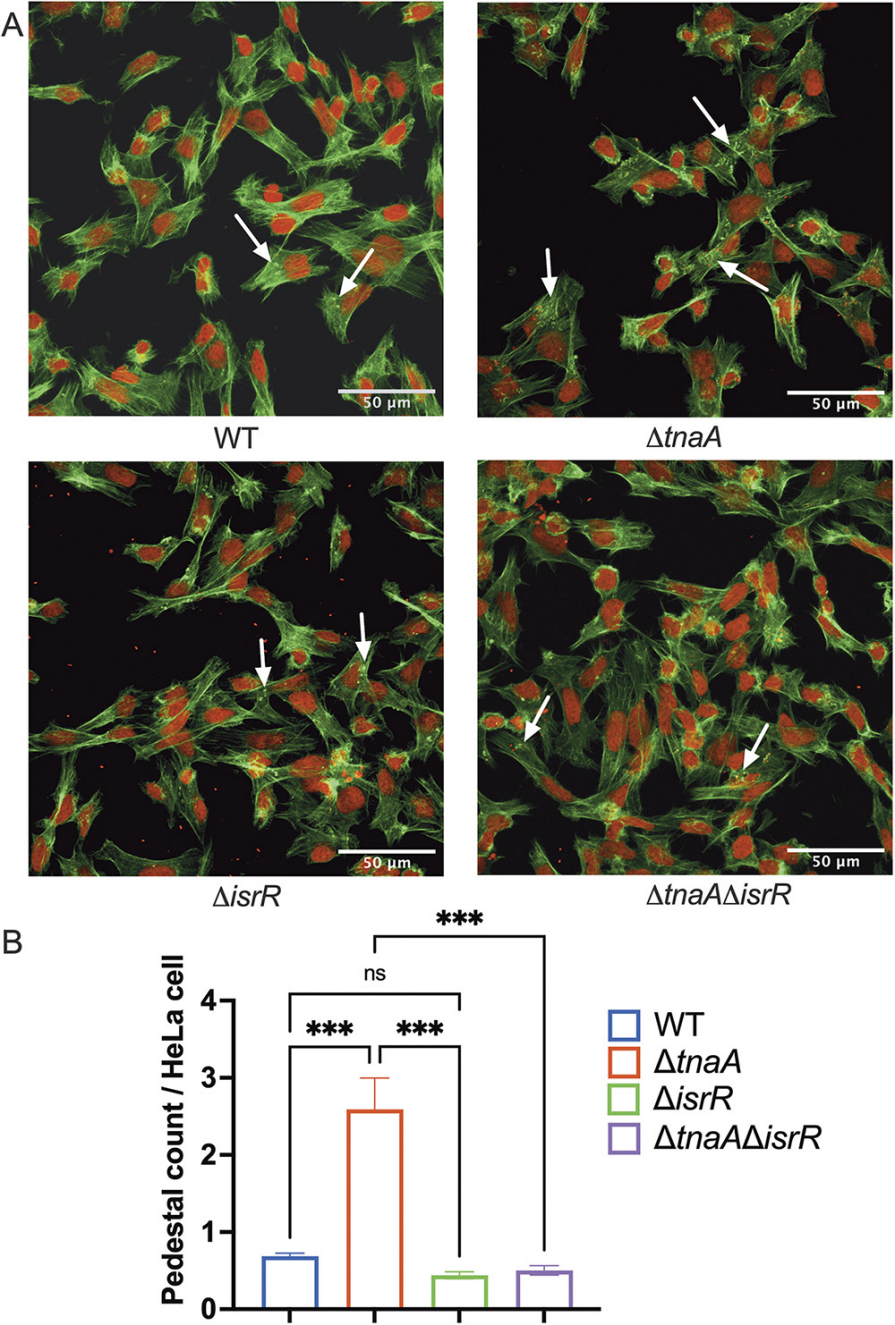
IsrR triggers pedestal formation in the absence of indole. (A) Fluorescein actin staining analysis. HeLa cells were infected with WT EHEC, Δ*tnaA* EHEC, Δ*isrR* EHEC, or Δ*tnaA*Δ*isrR* EHEC. At 5 h postinfection, cells were washed and stained with FITC-phalloidin to visualize actin (green) and propidium iodide to stain for bacteria and nuclei (red). Pedestals were visualized as green puncta (white arrows). Scale bars, 50 μm. (B) Quantitative analysis on the number of pedestals per HeLa cell. Pedestals were enumerated for each field, with each field containing approximately 20 cells. The number of pedestals per infected cell was quantified (*n *=* *3). *P*-value was calculated using one-way ANOVA followed by Bonferroni’s multiple-comparison test. Error bars represent standard deviations. ***, *P < *0.001; ns, not significant.

The LEE-dependent AE lesion formation is a hallmark of EHEC infection. EHEC attaches to the host cells by remodeling the actin cytoskeleton forming a pedestal-like structure (6). These pedestals can be visualized using fluorescein actin staining assays, where the actin is stained in green and EHEC cells and cell nuclei are stained in red. The pedestals appear as green puncta beneath the red bacteria. Congruent with our qRT-PCR and Western blot phenotypes showing decreased LEE expression, we observed a decrease in pedestal formation in the double mutant Δ*tnaA*Δ*isrR* compared to Δ*tnaA* EHEC (Fig. 3A and B). There was not a significant difference in pedestal formation between WT and Δ*isrR* (Fig. 3A and B). Together, our results indicate that IsrR-dependent LEE induction is inhibited in the presence of endogenous indole, and IsrR acts as an activator of the LEE in its absence.

### Exogenous indole decreases LEE gene expression independently from IsrR.

**FIG 4:**
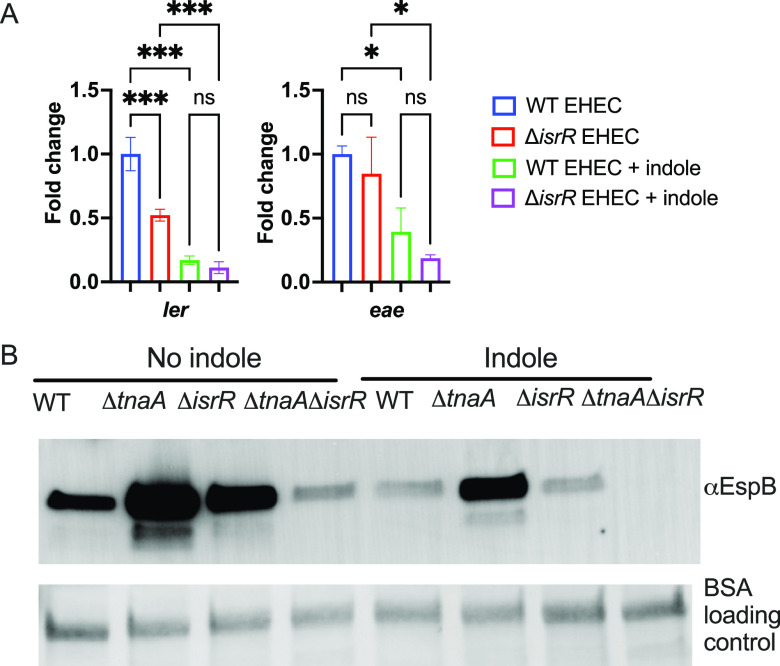
High concentration of exogenous indole affects the expression of virulence genes. (A) qRT-PCR analysis comparing expression of *ler* (master regulator of LEE pathogenicity island) and *eae* (intimin) from WT and Δ*isrR* EHEC in the presence and absence of 500 μM exogenous indole. One-way ANOVA followed by Bonferroni’s multiple-comparison test was used to calculate statistics. ***, *P < *0.001; *, *P* <0.05; ns, not significant. (B) Western blot on secreted protein EspB comparing WT EHEC, Δ*tnaA* EHEC, Δ*isrR* EHEC, or Δ*tnaA*Δ*isrR* EHEC in the presence or absence of indole. (A and B) Data are representative of at least three independent experiments. Fold change was calculated relative to an internal control *rpoA*. Error bars represent standard deviations.

Previously we have shown that a membrane-bound histidine kinase, CpxA, senses exogenous indole (2). To dissect indole signaling through the CpxA-CpxR two-component system from IsrR, we treated WT and Δ*isrR* EHEC with indole. We observed that indole treatment decreased the expression of virulence genes in both WT and Δ*isrR* (Fig. 4A). Similarly, T3SS protein (EspB) secretion was reduced on indole treatment in WT and all mutant strains (Fig. 4B). This suggested that the CpxA-CpxR indole signaling node, which senses exogenous sources of this compound, remained active in the mutant strains, and therefore IsrR may act independently of CpxA to regulate the expression of virulence genes. Because EHEC can produce indole and an endogenous pool of indole is present locally in the bacterial cytoplasm to interact with IsrR, it is conceivable that IsrR-dependent regulation becomes important while fine-tuning virulence gene expression under low indole conditions (Fig. S3).

10.1128/mbio.01939-22.3 FIG S3 Working model of the role of indole sensing regulator (IsrR) in indole signaling. A high concentration of exogenous indole allows indole to diffuse through the outer membrane where it is sensed by a membrane-bound histidine kinase, CpxA, which regulates the expression of virulence genes through CpxR. Exogenous indole can reach the cytoplasm through diffusion and can directly interact with IsrR. WT EHEC produces indole and the presence of the pool of endogenous indole inhibits IsrR activation of virulence genes. In the absence of indole, IsrR acts as an activator of LEE genes allowing IsrR to fine-tune the expression of virulence genes in variable indole conditions. Download FIG S3, PDF file, 0.1 MB. Copyright © 2022 Kumar et al. 2022 Kumar et al. https://creativecommons.org/licenses/by/4.0/ This content is distributed under the terms of the Creative Commons Attribution 4.0 International license.

### IsrR increases C. rodentium pathogenesis.

**FIG 5:**
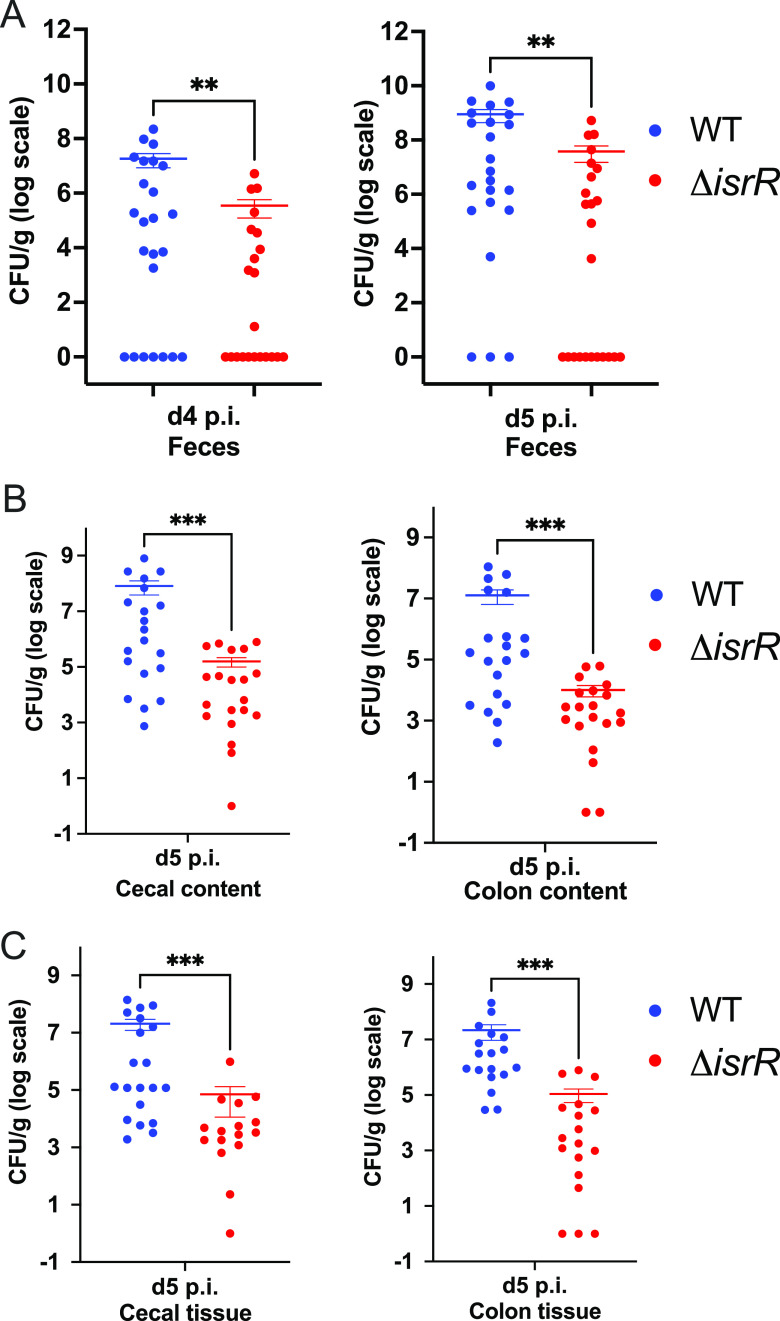
C. rodentium has a fitness defect in the absence of IsrR. Four- to six-week old C3H/HeJ mice were infected with WT or Δ*isrR* C. rodentium. Pathogen burden was enumerated at indicated time points. (A) C. rodentium loads recovered from feces on day 4 and day 5 postinfection (p.i.). Mice were sacrificed on day 5 and bacterial loads were enumerated from (B) contents of cecum and colon, and (C) cecal and colon tissues. (A–C) Groups were compared using the nonparametric Mann-Whitney *U* test. **, *P < *0.01; ***, *P < *0.001. Each data point represents a sample from an individual mouse.

**FIG 6:**
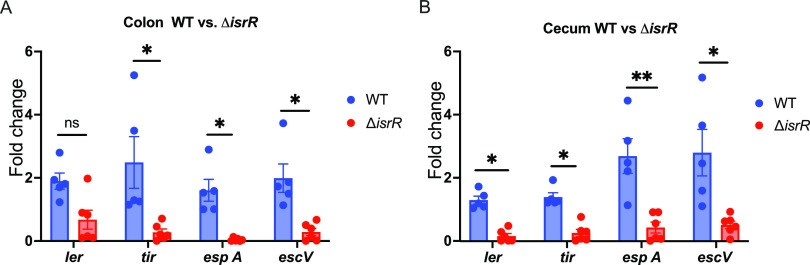
IsrR activates virulence genes *in vivo*. Four- to six-week-old C3H/HeJ mice were colonized with WT or Δ*isrR* C. rodentium. Mice were sacrificed on day 5 postinfection. (A) Colon and (B) cecum contents were collected, and the expression of virulence genes encoded in the LEE pathogenicity island was analyzed. Groups were compared using the nonparametric Mann-Whitney *U* test and followed by multiple-correction using the Bonferroni-Dunn method. *, *P < *0.05; **, *P < *0.01, ns, not significant. Each data point represents a sample from an individual mouse.

To determine the role of IsrR during murine infection, we utilized C. rodentium, a surrogate model to study EHEC pathogenesis, as EHEC cannot infect mice. Notably, WT C. rodentium lacks *tnaA* and cannot produce indole (2). This allowed us to directly utilize WT C. rodentium as a proxy for Δ*tnaA* EHEC to understand the function of IsrR during infection in the absence of endogenous indole. Indole decreases LEE gene expression of C. rodentium *in vitro*, (Fig. S4A), mimicking the phenotype of the Δ*isrR and* Δ*tnaA* Δ*isrR* EHEC mutants that still respond to exogenous sources of indole (Fig. 4). This mutant was complemented in *trans* with *isrR* in an arabinose-inducible system, which was the only system that allowed us to clone and express this gene to address any polar effects, even though isrR is not encoded within an operon and is a standalone gene. We note that arabinose interferes with IsrR-dependent virulence gene expression in C. rodentium. Overexpression of IsrR in the Δ*isrR* C. rodentium strain leads to an increase in the virulence gene expression even when compared to WT, which further supports our hypothesis that IsrR acts as an activator of virulence genes (Fig. S4B). In agreement with our *in vitro* results with EHEC and C. rodentium, Δ*isrR* C. rodentium is attenuated for infection in mice. Mice infected with Δ*isrR* present decreased pathogen burden in feces (Fig. 5A), colon and cecum contents (Fig. 5B), as well as colon and cecum tissues (Fig. 5C) compared to WT infected animals. Additionally, mice infected with Δ*isrR* C. rodentium displayed a delay in mortality (Fig. S5). Moreover, we observed reduced levels of LEE gene expression (*ler*, *tir*, *espA*, and *escV*) by Δ*isrR* C. rodentium compared to WT in the colon and cecum of these animals (Fig. 6A and B). Hence, our results indicate that IsrR plays an important role during *in vivo* fitness and the pathogenesis of C. rodentium in the gut.

10.1128/mbio.01939-22.4 FIG S4 Exogenous indole decreases virulence gene expression of C. rodentium *in vitro*. (A) qRT-PCR analysis comparing expression of virulence-related genes in the WT and Δ*isrR* C. rodentium DBS770 strains in the presence of 500 μM indole. *P*-value is calculated using two-way ANOVA followed by Bonferroni’s multiple comparisons test. *, *P < *0.05; ns, not significant. (B) qRT-PCR analysis comparing expression of virulence-related genes in the WT, Δ*isrR* and Δ*isrR*::p*isrR* C. rodentium DBS770 strains in the presence of 500 μM indole and 0.1% L-Arabinose. pBAD isr-his-myc plasmid is used for complementation and induced with 0.1% L-Arabinose. *P*-value is calculated using two-way ANOVA followed by Bonferroni’s multiple comparisons test. *, *P < *0.05; ns, not significant. Download FIG S4, PDF file, 0.1 MB. Copyright © 2022 Kumar et al. 2022 Kumar et al. https://creativecommons.org/licenses/by/4.0/ This content is distributed under the terms of the Creative Commons Attribution 4.0 International license.

10.1128/mbio.01939-22.5 FIG S5 A modest increase in Shiga toxin expression in an IsrR deficient C. rodentium counteracts mice mortality phenotype. C3H/HeJ mice 4 to 6 weeks old were colonized with WT or Δ*isrR* C. rodentium. (A) Mice were sacrificed on day 5 post-infection and expression of Shiga-toxin (*stx*) from pathogen recovered from colon was measured. ns, not significant. Each data point represents a sample from an individual mouse. (B) Survival analysis of mice infected with WT or Δ*isrR* C. rodentium. N = 15 mice per group. Statistics were calculated with Log-rank (mantel-Cox) test. Download FIG S5, PDF file, 0.1 MB. Copyright © 2022 Kumar et al. 2022 Kumar et al. https://creativecommons.org/licenses/by/4.0/ This content is distributed under the terms of the Creative Commons Attribution 4.0 International license.

## DISCUSSION

Indole is an abundant small molecule present in the gut. Indole is synthesized by the microbiota that resides in the lumen, where its concentration is highest. Indole is absorbed by epithelial cells, and its concentration decreases at the surface of the epithelial lining (2, 3). This gradient in concentration is monitored by EHEC and C. rodentium to optimize LEE gene expression and niche colonization. Indole concentrations found in the lumen due to its production by the microbiota are sensed through the HK CpxA and decrease LEE gene expression in this intestinal compartment, which is unsuitable for colonization by these pathogens (2). However, it is clear that this signaling cascade is more complex, because a switch favoring LEE expression has to occur at the epithelial lining. Here, we add another player to the indole signaling cascade that controls virulence expression of these enteric pathogens. Our comprehensive approach consisting of several comparisons between WT and Δ*tnaA* EHEC (that does not produce endogenous indole) with or without exogenous indole helped us identify a novel indole sensing regulator (*isrR*), whose expression is increased in response to indole (Fig. 1). IsrR is an orphan σ^54^ RR, which are transcriptional activators, because a σ^54^RNA polymerase does not form an open complex and requires a σ^54^ RR to complete this process (16). The emerging scenario from our data suggest that IsrR can only activate LEE expression in the absence of endogenous indole (Fig. 2 and 3), and does not “sense” exogenous indole, given that LEE expression is decreased by this signal in a Δ*isrR* strain (Fig. 4). Indole moonlights as a signal and a metabolite, and under low exogenous indole conditions it is used as a metabolite. This would free IsrR and allow it to activate LEE gene expression in EHEC, possibly at the low indole concentrations present at the surface of the epithelial cells, where the deployment of the type III secretion system is desired. The observation that exogenous indole, purportedly produced by the lumen microbiota, enhances expression of *isrR* (Fig. 1), may be the switch mechanism to prime EHEC to express the LEE and form AE lesions on enterocytes. This hypothesis is corroborated in the C. rodentium murine infections (Fig. 5 and 6). C. rodentium does not produce endogenous indole, and the C. rodentium *isrR* mutant is attenuated (Fig. 5 and 6). How IsrR-dependent LEE gene expression is responsive to indole, and whether IsrR directly promotes LEE gene expression are still open questions. It is also unknown whether IsrR interfaces with the CpxA HK at any level, given that it is an orphan RR. Because IsrR is a σ^54^ RR, it may connect this signaling cascade with the nitrogen-sensing NtrBC two-component system (16). Moreover, it can also interconnect with the QseEF two-component system, where QseF is also a σ^54^ RR. QseEF controls LEE gene expression and tryptophan metabolism at multiple transcriptional and posttranscriptional levels (17–20).

Here, we show that enteric pathogens discriminate sensing endogenous and exogenous indole through different regulators within this signaling cascade. They sense endogenously produced indole and adjust their virulence repertoire by utilizing an orphan indole sensing regulator (IsrR). In the presence of indole, IsrR remains inactive, while low/absence of indole allows IsrR to act as an activator of LEE genes (Fig. S3). Indole also acts as an autoinducer of the *tna* operon and therefore low indole concentrations decrease pool of endogenous indole, allowing IsrR to activate expression of virulence genes. Many GI pathogens such as Klebsiella, *Shigella*, and others encode the *isrR* gene, and may employ this strategy to fine-tune expression of their virulence genes within different intestinal microcompartments.

## MATERIALS AND METHODS

### Strains, plasmids, and culture conditions.

Strains and plasmids used in this study are listed in Table S1. WT EHEC O157:H7 strain 86-24, Citrobacter rodentium (DBS770) and their isogenic mutants were routinely grown in LB. To express the type III secretion system (T3SS), low glucose (1g/l) DMEM was used as these conditions have been shown to induce the T3SS (3). Bacterial cultures were grown anaerobically until the late log phase to an OD_600_ of 0.6 for all *in vitro* experiments. Anaerobic growth was performed using either the GasPak EZ anaerobe container system (Becton, Dickinson) or Bactron EZ anaerobic chamber (Sheldon Manufacturing). HeLa cells were routinely cultured in high glucose DMEM, defined as 4.5g/L glucose DMEM, 10% FBS, and penicillin + streptomycin + glutamine (PSG) cocktail.

10.1128/mbio.01939-22.6 TABLE S1 Strains and Plasmids. Download Table S1, DOCX file, 0.02 MB. Copyright © 2022 Kumar et al. 2022 Kumar et al. https://creativecommons.org/licenses/by/4.0/ This content is distributed under the terms of the Creative Commons Attribution 4.0 International license.

### Recombinant DNA techniques.

All primers used for mutant and plasmid construction can be found in Table S2.

10.1128/mbio.01939-22.7 TABLE S2 Primers. Download Table S2, DOCX file, 0.01 MB. Copyright © 2022 Kumar et al. 2022 Kumar et al. https://creativecommons.org/licenses/by/4.0/ This content is distributed under the terms of the Creative Commons Attribution 4.0 International license.

### Construction of deletion mutants of EHEC and Citrobacter rodentium.

Isogenic mutants of 86-24 EHEC were created using the λ red recombination technique (21). Briefly, pKD4 was used to generate the deletion PCR products. Strains harboring pKD46 were used to perform the recombination and pCP20 was used to resolve the insertions. Sequencing was performed to confirm all mutant strains.

### Western blot for secreted proteins.

Bacterial cultures were grown in low glucose DMEM anaerobically, and secreted proteins were isolated as previously described (2). Ten micrograms of bovine serum albumin (BSA) were added to secreted protein samples as a loading control. Secreted proteins were concentrated, separated on a 5–15% SDS-PAGE gel, transferred to polyvinylidene fluoride (PVDF) membrane, and blocked with 3% milk in PBS containing 0.05% Tween (PBST). Membranes were probed with either anti-EspB or anti-EspA primary antibody, washed, and then incubated with a secondary antibody conjugated to streptavidin-horseradish peroxidase. Invitrogen enhanced chemiluminescence (ECL) reagent was added, and the membranes were developed using the Bio-Rad ChemiDocTM Touch Imaging System (Software 1.0.0.15) with Image Lab 5.2.1 software for image display. Each experiment was repeated at least three times.

### RNA extraction and qRT-PCR.

Bacterial strains were grown in the absence or presence of indole (Sigma) to an OD_600_ of 0.6. RiboPure bacterial isolation kit was used to extract RNA from three biological replicates using the manufacturer’s protocols (Ambion). Quantitative real-time PCR (qRT-PCR) was performed as follows. Briefly, 2 μg of diluted extracted RNA was converted to cDNA with the addition of superscript, random primers, DTT, and dNTPs. Validated primers (Table S2) and SYBR green were added to the cDNA and the mix run in Quantstudio 6 Flex (Applied Biosystems). Data were collected using QuantStudio Real-Time PCR Software v1.3, normalized to endogenous *rpoA* levels, and analyzed using the comparative critical threshold (CT) method. One-way ANOVA was used when comparing three groups or more, followed by Bonferroni’s multiple hypothesis correction. A *P* value of <0.05 was considered significant.

### Fluorescein actin staining assays.

Assays were performed as described (22). Briefly, Confluent HeLa cells were grown overnight at 37°C, and 5% CO_2_ on coverslips in wells containing high glucose (4.5g/l) DMEM. Late log phase bacterial cultures with equal CFU grown in low glucose DMEM for 5 h were diluted 100:1 (bacteria to DMEM) to infect HeLa cells. After 5 h of infection, the coverslips were washed, fixed, and permeabilized. The samples were treated with fluorescein Isothiocyanate (FITC)-labeled phalloidin to visualize actin accumulation and propidium iodide to visualize bacterial DNA and HeLa nuclei, respectively. The coverslips were then mounted on slides and imaged with a confocal microscope. The number of bacteria attached per HeLa cell was quantified. Replicate coverslips from multiple experiments were quantified, and statistical analyses were performed using one-way ANOVA followed by Bonferroni’s multiple-comparison test.

### Murine infections.

C3H/HeJ mice were purchased from The Jackson Laboratory and housed in a specific pathogen-free facility at UT Southwestern Medical Center. All experiments were performed under IACUC approved protocols. At 3 to 4 weeks of age, female C3H/HeJ mice were infected with WT or isogenic Δ*isrR* C. rodentium. Fecal pellets were collected over time and mice were sacrificed on day 5 to collect colon and cecum contents as well as colon and cecum tissues to enumerate bacteria present in content and attached bacteria. Samples were resuspended in PBS, normalized to feces weight, and were plated on appropriate antibiotics for colony counting. The statistical comparison between groups was performed using the unpaired Mann-Whitney *U* test.

### Tissue collection, RNA isolation, and qRT-PCR.

Mice were sacrificed on day 5 postinfection and the colon tissue and content were collected. The tissue was washed in PBS twice to remove any residual fecal content. The content and tissues were snap-frozen in liquid nitrogen and stored at −80°C until use. RNA was isolated from individual mice fecal pellets using the RNeasy Power Microbiome kit (Qiagen) as per the manufacturer’s instructions. qRT-PCR was performed as described earlier using Quantstudio 6 Flex (Applied Biosystems). *rpoA* was used as an internal control for Citrobacter rodentium. Significance was determined by nonparametric Mann-Whitney *U* test, and multiple corrections were performed using the Bonferroni-Dunn method.

### RNA sequencing and analysis.

Briefly, RNA extracted as described above was used to perform RNA sequencing experiments. RNA isolated from three replicates was sent for RNA sequencing at UT Southwestern Medical Center Next Generation Sequencing Core. RNA libraries were prepared using Illumina ScriptSeq Complete Kit (Bacteria) (Catalog no. BB1224). RNA libraries were run on Illumina HiSeq 2500 sequencer with SE-50. To analyze the data, (23) reads were mapped to the Escherichia coli O157:H7 str. Sakai genome using Bowtie2. The number of reads of each gene was determined using the featureCounts package and differential expression was analyzed using DESeq2 (23).

### Quantification and statistical analysis.

The statistical tests and sample sizes are present within each figure legend. Generally, *P-*values were calculated using one-way ANOVA with Bonferroni multiple comparison posttest when 3 or more experimental groups were compared. All enumeration of bacteria by serial dilution and plating was log-transformed to normalize the data. For mice experiments, *P-value*s were calculated using Mann-Whitney *U* test when 2 experimental groups were compared followed by Dunn’s posttest for correction. Statistics for survival analysis was calculated using the log rank (Mantel- Cox) test. For all *in vitro* experiments, error bars represent standard deviation. For *in vivo* experiments, error bars represent the standard error of mean. RNA seq analysis and data visualization were carried out in R v4.1.2. as described above. A *P-*value of < 0.05 was considered statistically significant. All statistical tests were performed using Prism 9 v9.3.1 (GraphPad Software, LLC). Statistical significance was defined as follows: *, *P* < 0.05; **, *P* < 0.01; ***, *P* < 0.001.
