# Supplementary figures and images for: Indole Sensing Regulator (IsrR) Promotes Virulence Gene Expression in Enteric Pathogens
Source: mBio. 2022 Aug 2;13(4):e01939-22. doi: 10.1128/mbio.01939-22 (PMC9426417; doi:10.1128/mbio.01939-22)

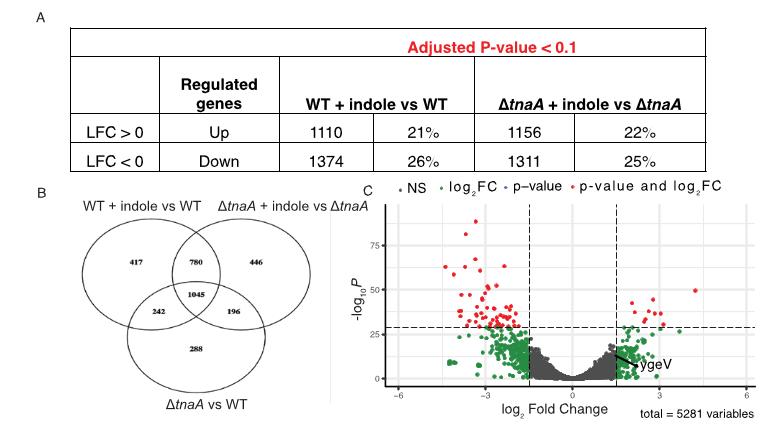

Supplement: FIG S1 [file mbio.01939-22-sf001.tif]

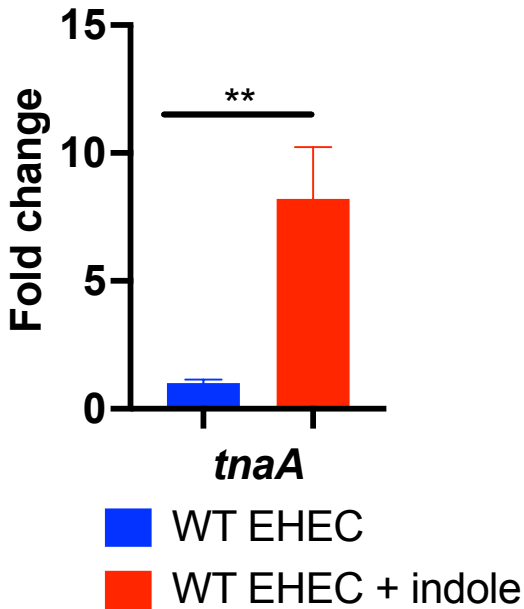

Supplement: FIG S2 [file mbio.01939-22-sf002.pdf]

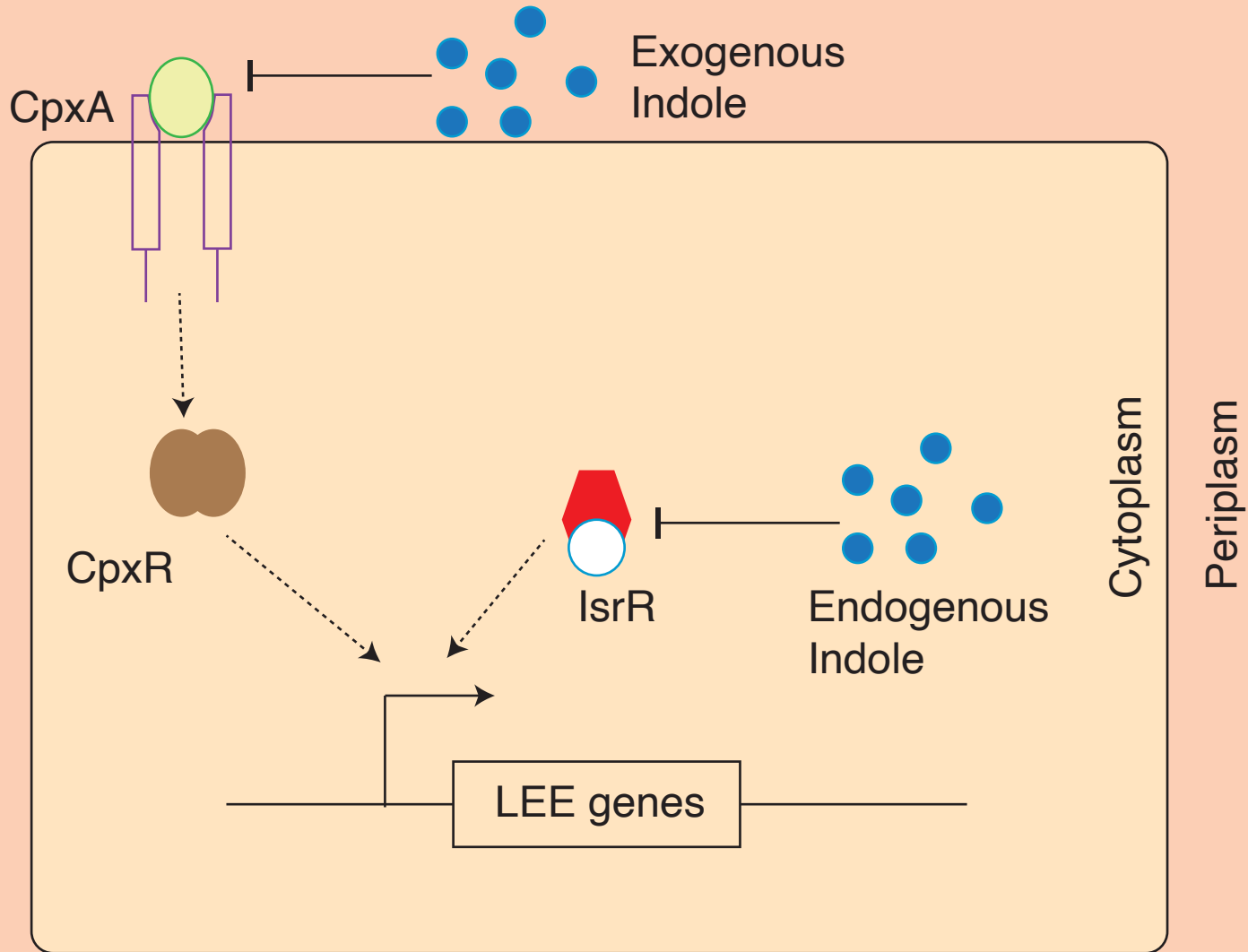

Supplement: FIG S3 [file mbio.01939-22-sf003.pdf]

A

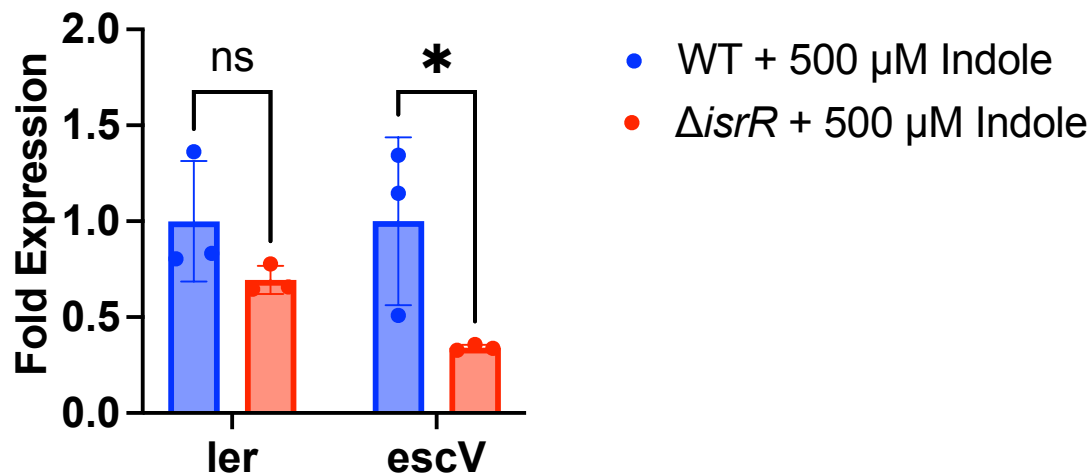

B

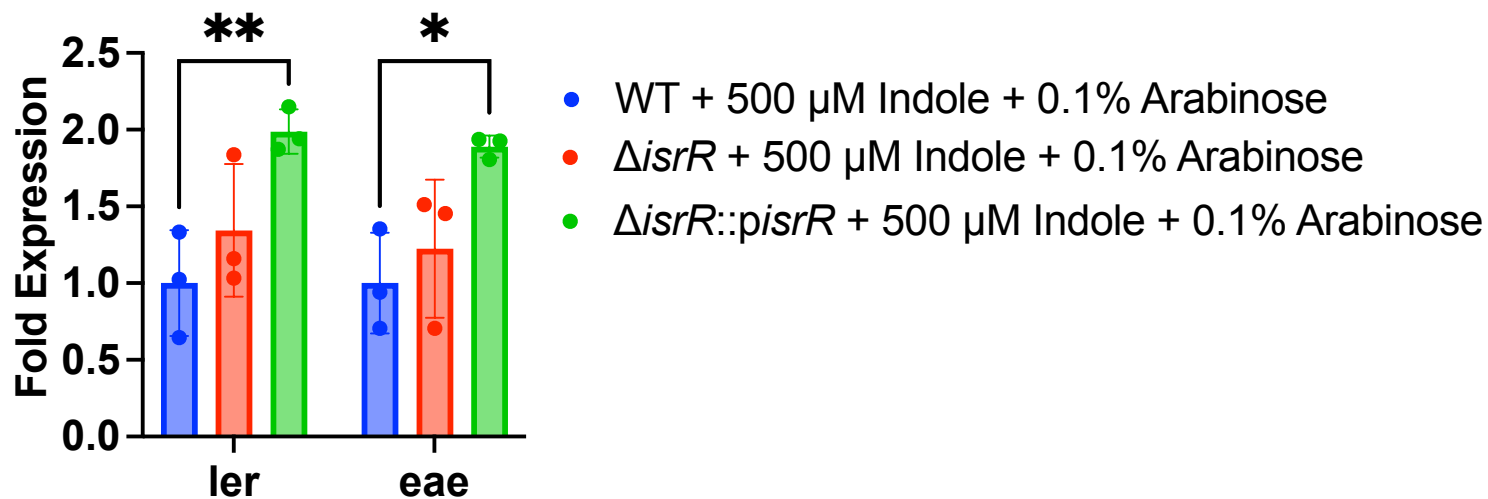

Supplement: FIG S4 [file mbio.01939-22-sf004.pdf]

A

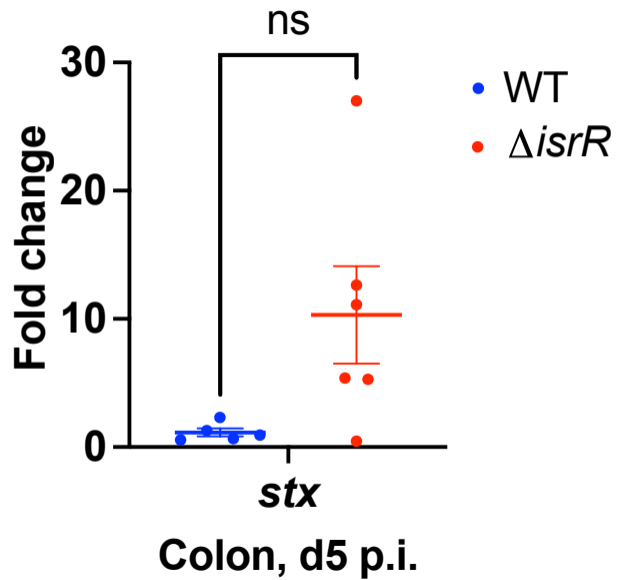

B

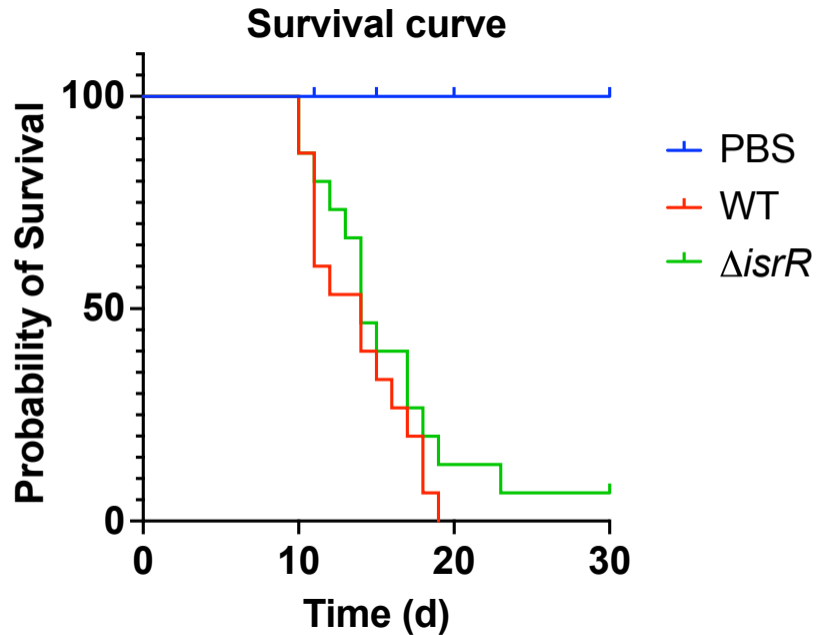

Supplement: FIG S5 [file mbio.01939-22-sf005.pdf]
