# Supplementary material for: Indole Sensing Regulator (IsrR) Promotes Virulence Gene Expression in Enteric Pathogens
Source: mBio. 2022 Aug 2;13(4):e01939-22. doi: 10.1128/mbio.01939-22 (PMC9426417; doi:10.1128/mbio.01939-22)
Supplement: TABLE S1 [file mbio.01939-22-st001.docx]

**Table S1. Strains and Plasmids**

| Strains |  |  |
| --- | --- | --- |
| Strain | Description | Reference |
|  | EHEC |  |
| WT EHEC | *E. coli* O157:H7 86-24 Clinical isolate | (24) |
| △*tnaA* | isogenic *tnaA* deletion mutant | (2) |
| △*isrR* | isogenic *isrR* deletion mutant | This study |
| △*tnaA*△*isrR* | isogenic *tnaA* and *isrR* double deletion mutant | This study |
|  | *Citrobacter rodentium* |  |
| DBS770 | *C. rodentium* | (25) |
| △*isrR* DBS770 | isogenic *tnaA* deletion mutant in *C. rodentium* | This study |
| Plasmids |  |  |
| pKD4 | λ red template plasmid | (21) |
| pKD46 | λ red helper plasmid | (21) |
| pCP20 | λ red helper plasmid | (21) |
| pACYC177 | Cloning vector | New England Biolabs |
