# Supplementary material for: Indole Sensing Regulator (IsrR) Promotes Virulence Gene Expression in Enteric Pathogens
Source: mBio. 2022 Aug 2;13(4):e01939-22. doi: 10.1128/mbio.01939-22 (PMC9426417; doi:10.1128/mbio.01939-22)
Supplement: TABLE S2 [file mbio.01939-22-st002.docx]

**Table S2. Primers**

| **primer** | **Sequence 5’ to 3’** |
| --- | --- |
|  | **qRT-PCR EHEC** |
| *rpoA* F | GTGACCCTTGAGCCTTTAGAG |
| *rpoA* R | ACACCATCAATCTCAACCTCG |
| *espA* F | AGCTATTTGAGGAACTCGGTG |
| *espA* R | CATCTTTTGTGCCGTGGTTG |
| *espB* F | GGTCAAGGCTACGGAAAGTG |
| *espB* R | TCTTCAGCAAAGTCAGAGGC |
| *tir* F | GAGGGAGTCAAATAGCGGTG |
| *tir* R | ATCTGAACGAAGGCTGGAAG |
| *eae* F | TGGGATGTTCAACGGTAAGTC |
| *eae* R | TTTAACCTCAGCCCCATCAC |
| *Ier* F | CGAGAGCAGGAAGTTCAAAGTG |
| *ler* R | ACACCTTTCGATGAGTTCCG |
| *escV* F | GAGTGCAAAAGGAAAGCCAG |
| *escV* R | ATGATACCAGCAATAGCGTCC |
| *ygeV* F | GTAAAACAGGGTTGATTCAGGC |
| *ygeV* R | CGACTTGTATTGGGCTACTGG |
| *tnaA* F | AGGGATTAGAACGCGGTATTG |
| *tnaA* R | CGGAGTTACTGGTGATGGTTG |
|  | **qRT-PCR DBS770** |
| *espA* F | ACGAGGTAACAACCATGCGAGTGT |
| *espA* R | CTGCCTGGCATTGCTTTCCAGAAT |
| *tir* F | ATCAGATATCTCGCAAGCTCG |
| *tir* R | CAACTCCATCTCCCATTCCTG |
| *eae* F | TGCGAAAGATACAGCCCTTAG |
| *eae* R | ACCTCTGCCGTTCCATAATG |
| *rpoA* F | ACGTCAGCCGGAAGTGAAAGAAGA |
| *rpoA* R | AGCGGACAGTCAATTCCAGATCGT |
| *ygeV* F | CGGGAGCAGATAATGGGTAAAG |
| *ygeV* R | AGCCATTGATCCTGAACCG |
|  |  |
|  |  |
| *escV* F | GGGCGATGAAGTTTGTAAAAGG |
| *escV* R | CCAACCGACAATACAGAAAACAG |
|  | **Primers for cloning and mutations** |
|  |  |
| LR_ ygeV_EHEC_F | TCAGTATTGATGCAAATTCAACCGACAATTCAGCGTTTTGCCAGAATGCTTGCCAGCG |
| AK_ygeV_EHEC*_R* | TGTGTTTAACAACTCATATTTCTTAATCTTGCGATAGAGCGTAGCAATGCCGATGCC |
| AK_ygeV_CR_F | CTGATGCAAATCCAGCCTAAAATTCAGAAATTTGCGAGGATGCTTTCCAGCGTGCTC |
| AK_ygeV_CR_R | GCGATACAACGTGGCAATGCCAATACCTAATTCGCTGGCCACCTGTTTTTTATTAC |
